# Fibromax-based nonalcoholic fatty liver disease in chronic obstructive pulmonary disease patients with obstructive sleep apnea: Methodological considerations

**DOI:** 10.12688/f1000research.12581.1

**Published:** 2017-09-08

**Authors:** Denis Monneret

**Affiliations:** 1 Department of Metabolic Biochemistry, La Pitié Salpêtrière-Charles Foix University Hospital (AP-HP), Paris, F-75013, France

**Keywords:** nonalcoholic fatty liver disease, chronic obstructive pulmonary disease, obstructive sleep apnea, Fibromax, biomarker standardization

## Abstract

The relationship between nonalcoholic fatty liver disease (NAFLD) and obstructive sleep apnea (OSA) has been well demonstrated, but remains to be evidenced in chronic obstructive pulmonary disease (COPD). Recently, Viglino *et al.* (Eur Respir J, 2017) attempted to determine the prevalence of liver fibrosis, steatosis and nonalcoholic steatohepatitis (NASH) in COPD patients, some of whom had OSA, basing the NAFLD diagnostic on three circulating biomarker-based liver scores: the FibroTest, SteatoTest and NashTest, from the Fibromax® panel. Among the main findings, the absence of OSA treatment emerged as independently associated with liver fibrosis and steatosis, when compared to effective treatment. However, besides the low number of treated patients, no polysomnographic respiratory data was provided, making it difficult to differentiate the impact of OSA from that of COPD in NAFLD prevalence. Furthermore, NAFLD diagnosis relied exclusively on circulating biomarker-based liver scores, without histological, imagery or other liver exploratory methods. Therefore, in this article, some methodological points are reminded and discussed, including the choice of OSA measurements, and the significance of ActiTest and AshTest scores from Fibromax® in this pathophysiological context.

The relationship between nonalcoholic fatty liver disease (NAFLD) and obstructive sleep apnea (OSA) has been well demonstrated ^1– 3^, but remains to be evidenced in chronic obstructive pulmonary disease (COPD). To this end, using biomarker-based Fibromax® scores, Viglino *et al*. recently attempted to determine the prevalence of liver fibrosis, steatosis and nonalcoholic steatohepatitis (NASH) in COPD patients, which they found at nearly 61%, 41%, and 37%, respectively ^4^. Interestingly, the absence of OSA treatment emerged as independently associated with liver fibrosis and steatosis, when compared to effective treatment. However, the number of treated patients was low (10 *versus* 38 untreated), and no polysomnographic respiratory data was provided, making it difficult to differentiate the impact of OSA from that of COPD in NAFLD prevalence. Furthermore, NAFLD diagnosis relied exclusively on circulating biomarker-based liver scores, without histological, imagery or other liver exploratory methods. It is, therefore, the opportunity to remind and discuss some methodological points, especially concerning the choice of OSA measurements, and the significance of ActiTest and AshTest scores from Fibromax® in this pathophysiological context.

## 1) Impact of OSA on NAFLD in COPD patients

Recently, Pépin’s team showed a prevalence of liver steatosis of about 40–45% in moderate-to-severe OSA patients, with nearly 40–60% of patients displaying borderline NASH ^5^. They also showed in obese OSA patients that fibrosis and NAFLD-related lesions, like hepatocyte ballooning and lobular inflammation, were more severe in those with the highest nocturnal oxygen desaturation ^6^. Accordingly, nocturnal time spent at <90% oxygen saturation was independently associated with liver fibrosis in patients with suspicion of OSA ^7^. In the Viglino *et al.* study, the absence of OSA treatment emerged as an independent factor of liver fibrosis and steatosis, as compared to treatment. Including the absence *versus* effective OSA treatment in the multivariate model is not the most appropriate criterion, since the authors recently showed that 6–12 weeks of CPAP treatment did not reduce steatosis, NASH or liver fibrosis ^5^. This is in addition to the very low number of treated patients (10 *vs* 38 untreated). Instead, and according to the increasingly obvious hypothesis of chronic intermittent hypoxia (CIH) on NAFLD, the authors could have chosen the oxygen desaturation index as a criterion (e.g. with a cut-off < or ≥15 events/h), which they elsewhere claimed to be “ *a good marker of CIH*” ^6^. Studies displaying detailed results for both OSA and liver scores are few to date, and do not allow an in-depth analysis of their relationships. Therefore, in such NAFLD/COPD/OSA-related studies, polysomnographic respiratory profiles should be provided, including oxygen desaturation measurements, along with liver scores and detailed biology, for the overall COPD group and for OSA patients (all, treated and untreated), in order to strengthen conclusions and enable comparison with further studies.

## 2) Fibromax® scores

Viglino *et al.* focused on the three most appropriate NAFLD scores (i.e. FibroTest, SteatoTest, and NashTest). However, they did not provide ActiTest, another Fibromax® score proposed for the estimation of liver necroinflammatory activity in chronic hepatitis C and B ^8^, which is based on the measurement of the five Fibrotest® parameters *plus* alanine aminotransferase. Interestingly, ActiTest has been shown as highly accurate for the diagnosis of NASH and steatosis in patients with severe obesity, with notably an excellent negative predictive value for NASH at 96% using a cut-off at 0.29 ^9^. In another study on patients with suspected NAFLD, ActiTest showed a significant diagnostic value for NASH, which was not shown for FibroTest ^10^. The informative value of ActiTest on the inflammatory component of NAFLD in COPD patients with or without OSA remains questionable; it could therefore be provided and discussed with regards to other inflammatory biomarkers (tumour necrosis factor-α and leptin in the Viglino *et al*. study).

Furthermore, alcohol consumption at ≥20g/day (women) and ≥30g/day (men) was chosen by the authors as exclusion criteria to discard potential alcoholic steatohepatitis (ASH). However, alcohol consumption may vary over time, even in abstainers or occasional drinkers, and may thus introduce a misclassification bias ^11^. Self-reported alcohol consumption remains subjective and should ideally be evaluated using a reliable and objective measure. In this way, the AshTest score –the fifth of Fibromax® – is proposed for the detection of alcoholic steatohepatitis ^12^, and thus could be provided as a control for non-excessive alcohol consumption in such pathophysiological contexts.

## 3) Statistical consideration

Depending on the score, age, sex and/or weight and height are included in the Fibromax® calculation formulas. Therefore, the multivariate analyses from Viglino *et al*, which included age, sex and/or BMI as independent variables in addition to the Fibromax® score as a dependent variable may induce multicollinearity, and thus cause imprecise estimates of coefficient values or introduce large prediction errors in the case of extrapolation. Consequently, multicollinearity must be tested in such models, and controlled as much as possible.

## 4) Biomarker standardization

Viglino *et al.* did not provide any information about the methods used for Fibromax® parameters. Analytically, standardization allows the reduction of inter-laboratory variability. It is of particular importance for gamma-glutamyl transferase, known for its high inter-method variability, as well as for transaminases, which are measurable with or without pyridoxal 5-posphate as a coenzyme activator ^13^. Fibromax® proteins also need standardization given their weight in score calculation, especially α2-macroglobulin ^14^. Comparison with peer and method groups – through programs of quality control – allows inter-laboratory variation assessment; it is an analytical requirement of the ISO15189 standard for accreditation of medical laboratories, which is a strong guarantee of result reliability ^15, 16^. If ISO15189 certified methods are used for Fibromax® assays, it must be mentioned, along with methods and analyzers, in order to strengthen the biomarker component, to make it sufficiently informative to be compared with further studies.

To conclude, in such studies evaluating NAFLD, based exclusively on combined-biomarker scores without clinical, histological, imagery or other liver exploratory methods, information on assay methods, analyzers, and guarantees of analytical performance are required, which requires a strong collaboration between clinicians and lab practitioners.
