# Supplementary material for: Fibromax-based nonalcoholic fatty liver disease in chronic obstructive pulmonary disease patients with obstructive sleep apnea: Methodological considerations
Source: F1000Res. 2017 Sep 8;6:1669. [Version 1] doi: 10.12688/f1000research.12581.1 (PMC5710384; doi:10.12688/f1000research.12581.1)
Supplement: Peer review history

# Peer review: Referee response for version 1

**DOI:** 10.5256/f1000research.13625.r27720

**Published:** 2017-11-27

**Authors:** Omar A Mesarwi

**Affiliations:** 1 Division of Pulmonary, Critical Care, and Sleep Medicine, UC San Diego Department of Medicine, La Jolla, CA, USA

This article, by Dr. Monneret, presents many very salient points in reference to the article by Viglino et al. I would like to make a minor remark:

 In section 1, I think the article may benefit from a very brief mention of the biological differences between intermittent and sustained hypoxia - experimentally these exposures yield very different metabolic outcomes, and the mechanisms which underlie these effects (sympathetic activation, oxidative stress, tissue oxygen profiles, etc.) may be quite different in sustained hypoxia as in COPD, versus chronic intermittent hypoxia, as in OSA.

I have read this submission. I believe that I have an appropriate level of expertise to confirm that it is of an acceptable scientific standard.

# Peer review: Referee response for version 1

**DOI:** 10.5256/f1000research.13625.r27106

**Published:** 2017-11-27

**Authors:** Valentina Giorgio

**Affiliations:** 1 Fondazione Policlinico Universitario A. Gemelli, Università Cattolica Sacro Cuore, Institute of Pediatrics, Rome, Italy

This letter is interesting, written in good and fluent English, clearly addressing the limits of a paper attempting to find a link between obstructive sleep apnea (OSA) and Non alcoholic Fatty Liver, in patients with chronic obstructive pulmonory disease (COPD). I agree with Monneret that one of the main limit of this paper, among others, is that it is quite difficult to differentiate the impact of OSA from that of COPD in fatty liver prevalence, because of the common respiratory nature of OSA and COPD and the common negative effect in oxygen saturation. Therefore, although the paper has the merit of pointing out that non alcoholic fatty liver disease can be present -and maybe more often present- in patients with COPD, more  polysomnographic respiratory profiles should be provided, including oxygen desaturation measurements.

I have read this submission. I believe that I have an appropriate level of expertise to confirm that it is of an acceptable scientific standard.
